# Radiotracers for Imaging of Fibrosis: Advances during the Last Two Decades and Future Directions

**DOI:** 10.3390/ph16111540

**Published:** 2023-11-01

**Authors:** Olof Eriksson, Irina Velikyan

**Affiliations:** 1 Science for Life Laboratory, Department of Medicinal Chemistry, Uppsala University, 751 83 Uppsala, Sweden; irina.velikyan@akademiska.se; 2 Antaros Tracer AB, Dragarbrunnsgatan 46, 2 tr, 753 20 Uppsala, Sweden; 3 Nuclear Medicine and PET, Department of Surgical Sciences, Uppsala University, 752 85 Uppsala, Sweden

**Keywords:** fibrosis, fibrogenesis, fibrolysis, radiochemistry, collagen, positron emission tomography, molecular imaging

## Abstract

Fibrosis accompanies various pathologies, and there is thus an unmet medical need for non-invasive, sensitive, and quantitative methods for the assessment of fibrotic processes. Currently, needle biopsy with subsequent histological analysis is routinely used for the diagnosis along with morphological imaging techniques, such as computed tomography (CT), magnetic resonance imaging (MRI), and ultrasound (US). However, none of these imaging techniques are sufficiently sensitive and accurate to detect minor changes in fibrosis. More importantly, they do not provide information on fibrotic activity on the molecular level, which is critical for fundamental understanding of the underlying biology and disease course. Molecular imaging technology using positron emission tomography (PET) offers the possibility of imaging not only physiological real-time activity, but also high-sensitivity and accurate quantification. This diagnostic tool is well established in oncology and has exhibited exponential development during the last two decades. However, PET diagnostics has only recently been widely applied in the area of fibrosis. This review presents the progress of development of radiopharmaceuticals for non-invasive detection of fibrotic processes, including the fibrotic scar itself, the deposition of new fibrotic components (fibrogenesis), or the degradation of existing fibrosis (fibrolysis).

## 1. Introduction

### 1.1. Fibrosis in Health and Disease

Deposition of extracellular matrix (ECM) and, in particular, collagen in response to injury is critically important for wound healing in normal physiology. However, the excess of collagen deposition in such vital organs as liver, lung, heart, and kidney because of chronic diseases and inflammatory injuries may lead to tissue fibrosis, affecting the functionality of these organs [1,2,3,4,5]. The accumulation of collagen and ECM results in the loss of the tissue homeostasis and elasticity, and buildup of dysfunctional fibrotic tissue [4,6,7]. Sixteen different forms of collagens have been identified, with type 1 and 3 collagens as the most common ones.

The early stages of the fibrosis are often asymptomatic and painless, and disease progresses considerably by the time it can be detected using conventional diagnostic methods. Importantly, as the outcome of late-stage fibrosis can be lethal, diagnosis in the early stage of the disease development is crucial not only for the progression prediction and treatment planning, but also for monitoring response to the therapy. Despite the rise in incidence of fibrosis-related diseases, effective treatment options are currently limited [8]. Thus, there are ongoing efforts within the pharmaceutical industry to identify suitable targets, as well as drug molecules, to reduce fibrotic burden in tissue.

Conventional invasive diagnostic methods require tissue sampling, which can often present errors and side effects [9,10]. Moreover, multiple biopsies for the determination of disease progression and treatment response monitoring are rarely possible in clinical practice. There has been great interest in identification of suitable peripheral plasma biomarkers to reflect fibrosis degree. Recent and promising approaches include Pro-C3, a blood marker that may indicate ongoing aberrant overproduction of collagen (fibrogenesis) [11]. However, peripheral biomarkers, including the ones mentioned above, do not exhibit tissue specificity, and instead provide insights into the overall health status of all tissues in the body. Thus, tissue-specific and sensitive biomarkers of fibrosis would potentially provide great value in both tracking the effectiveness of intervention, as well as early diagnosis and treatment selection.

Non-invasive morphological imaging is provided by various ultrasound (US) technologies, magnetic resonance imaging (MRI), and computed tomography (CT) [2,12,13,14]. These technologies are widespread in clinical routine and provide high-resolution images of internal organ features, usually without the need for contrast agents. In particular, magnetic resonance elastography (MRE) and transient elastography (US) are useful for the detection of high-degree fibrosis in liver, and dual-energy CT is beneficial for lung examination. US and MRI do not use ionizing radiation and can thus be used safely also in young or vulnerable populations for repeated assessments. However, neither US, MRI, nor CT provide information on fibrogenesis and fibrolysis. Moreover, they are too insensitive to detect low-degree fibrosis and molecular changes in tissue, e.g., expression of particular receptors or enzymes. Molecular changes are expected to occur within shorter timeframes than macroscopic changes, and thus, molecular imaging might provide faster readout and earlier diagnosis of fibrotic lesions.

The unmet medical need for early non-invasive, sensitive, specific, and quantitative diagnosis of fibrosis and fibrosis-associated molecular processes motivated development of radiopharmaceuticals for nuclear molecular imaging using such technologies as single-photon emission tomography (SPECT) and positron emission tomography (PET) [9,15,16,17,18,19,20]. As mentioned above, the staging of the disease, as well as early and accurate monitoring of disease progression, is crucial for the efficiency of the treatment. The development of novel anti-fibrotic drugs is another aspect of such agent application, wherein phase 0 clinical studies under the microdosing concept [21,22,23,24,25,26] can be conducted to investigate the pharmacokinetics of a drug by isotopic radiolabeling, which could be of interest for decreasing the cost of the development. Furthermore, with the advent of therapeutical agents [27,28,29], it is highly topical to develop tissue-specific non-invasive biomarkers of fibrosis to investigate the drug action mechanism and to monitor the response to the treatment accurately and quantitatively. The increasing number of clinical studies for the development of molecular imaging agents is a positive indication of progress in the field [15,17,20,30].

### 1.2. Etiology of Fibrosis

**Figure 1:**
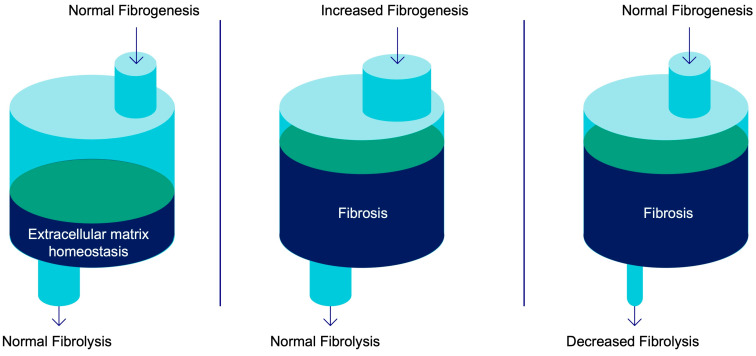
The amount of extracellular matrix (ECM) proteins in tissue is dependent on the balance of production of new collagen (fibrogenesis) and clearance of collagen fibers (fibrolysis). The scheme presents the extent of ECM content in tissue (the fibrotic scar), governed by the balance/dysregulation between fibrogenesis and fibrolysis.

The underlying biological mechanism of fibrosis formation is complex, and furthermore, the etiology differs depending on what tissue is affected. The amount of collagen in tissue is dependent on a delicate balance between the deposition of new collagen (“fibrogenesis”) and the degradation of collagen (“fibrolysis”) (Figure 1). The pathological accumulation of excess collagen in tissue (fibrotic scar) can thus be due to the dysregulation of either of the fibrogenic and/or fibrolytic processes. Whether fibrosis occurs due to increased fibrogenesis or decreased fibrolysis, the underlying pathological processes and the necessary steps for resolving fibrosis differ significantly. Thus, although detection of the fibrosis degree (actual amount of ECM deposition) in tissue is of high importance, it will not provide information on the ongoing change in fibrosis levels. In other words, a biopsy assessment of collagen concentration or an elastography measurement of stiffness tells us of the fibrosis grade at present, while an imaging marker of the fibrogenesis marker would tell us of the fibrosis grade tomorrow. This is especially important for the assessment of the treatment effect, as change in the fibrosis degree may be slow (months/years), while downregulation of fibrogenesis may happen relatively rapidly on the scale of days/weeks.

**Figure 2:**
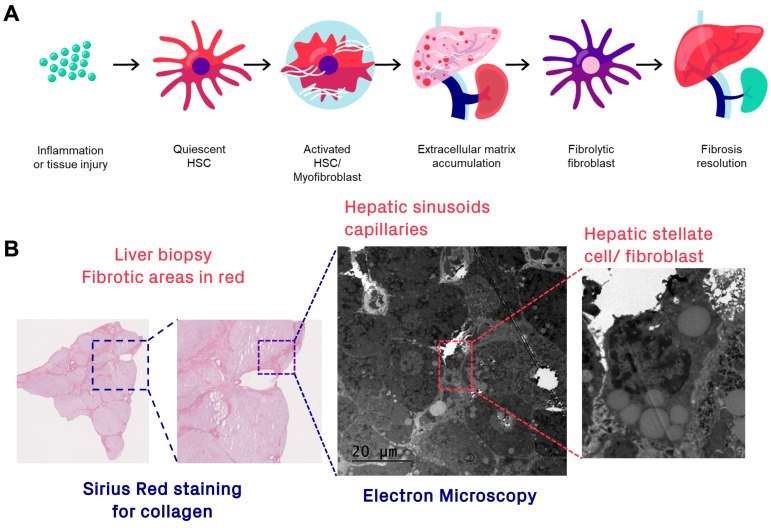
(**A**) Fibroblast involvement in ECM deposition and fibrosis resolution. (**B**) Example of activated HSC-mediated fibrosis development in liver.

The mechanisms of fibrogenesis and fibrolysis, fibrosis onset and resolution, are different depending on tissue and are not completely clear in many diseases. In liver fibrosis, the picture is quite clear though and serves as an illustrative example. The main cell type responsible for deposition of ECM in liver are the hepatic stellate cells (HSCs), the hepatic counterpart of the pericyte (Figure 2B). Quiescent HSCs are usually localized in the lining of the hepatic sinusoids (space of Disse). In normal physiology, HSCs serve to regulate the ECM content, crucially supporting the hepatic capillary bed. However, HSCs may be activated in response to acute hepatic injury or chronic inflammation and take on a myofibroblast-like phenotype (Figure 2A), which synthesizes and deposits collagen in the form of collagen fibrils and fibers (shown as Sirius red-positive areas in the hepatic biopsy in Figure 2B). The molecular basis for collagen processing has been described in detail previously [31]. Briefly, preprocollagen monomers are synthesized, post-translationally modified, and then form procollagen in triple-helix formation, which is released from the cytosol. Procollagen is further modified by enzymatic truncation of the N- and C-terminal domains to form tropocollagen, by, e.g., zinc metalloproteinases. Finally, multiple tropocollagens will crosslink to form collagen fibrils and subsequently collagen fibers, which is the main constituent of fibrotic lesions.

**Figure 3:**
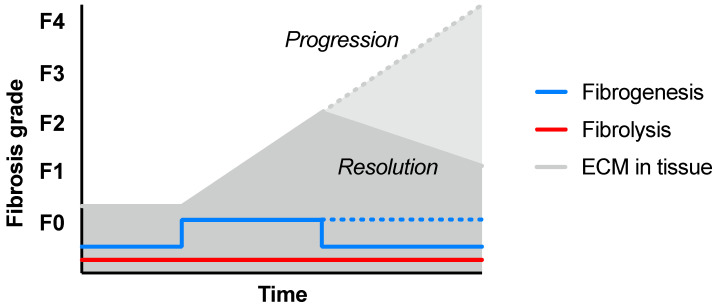
Theoretical progression or resolution of tissue fibrosis in response to difference in degrees of fibrogenesis and fibrolysis over time.

Chronic imbalance between pathologically upregulated fibrogenesis and insufficient ECM clearance could result in a gradually increasing fibrosis degree, with associated downstream complications, such as liver dysfunction or failure, cirrhosis, or cancer (Figure 3). However, the fibrosis grade can also be stabilized, reduced, or even resolved by normalization of fibrogenesis in combination with sustained fibrolysis (Figure 2A and Figure 3). Fibrolysis is a complicated process requiring several steps, including unwinding of the collagen fibrils and triple helices (by matrix metalloproteases), followed by enzymatic degradation of collagen monomer peptides (by, for example, fibroblasts expressing membrane-bound collagenases).

Thus, it is of interest in not only developing molecular imaging techniques to detect the actual fibrosis deposition (e.g., the collagen fibers in tissue, analogous to Sirius red staining of a biopsy), but also for surface markers of fibrogenic, as well as fibrolytic, cells.

Molecular imaging, e.g., of activated collagen-depositing myofibroblasts, is assumed to provide a non-invasive readout of the ongoing process of fibrogenesis in a tissue of interest, which is expected to change much faster than the change in the actual fibrosis degree, in response to treatment.

### 1.3. Publication Search and Selection

The literature search of studies published during the last two decades was limited to articles published in English, and was conducted using Scopus, PubMed/MEDLINE, Web of Science, Google Scholar, and SciFinder Scholar databases, as well as references of the retrieved articles, reviewing the titles and abstracts. Comment and errata letters, author reply letters, and conference abstracts were excluded. The number of the included publications was reduced after more careful review, keeping focus on the novel imaging agent development and validation, as well as clinical applications. This is a brief unstructured review on the publications from 2013 through 2023. The cutoff was used as development of radiotracers, for PET imaging of fibrosis is sparse before 2013. The aim of this review was to identify the complexity of the fibrosis physiology and, with that, the directions of the radiotracer development.

## 2. Imaging of Fibrogenesis and Fibrolysis

Visualization of fibrogenic or fibrolytic cells or processes conceivably requires detection of enzymes or surface receptors in low concentrations, and thus, nuclear imaging techniques are potentially required. MRI, CT, and US are most likely not sufficiently sensitive for such applications.

### 2.1. Targets and Radioligands to Detect Fibrogenesis and Fibrolysis

Several targets have been suggested as potential markers of fibrogenic or fibrolytic cells, and radioligand development for these are under active development (Table 1). Here, we will discuss the approaches that are either most researched, most promising, or furthest along in translation towards clinical development. We are aware that this list may be non-exhaustive.

#### 2.1.1. α_v_β_3_ and α_v_β_6_ Integrins

Integrins are a large family of proteins that facilitate interactions between cells and ECM. They are, furthermore, important for downstream signaling involving, for example, cellular differentiation and angiogenesis. Several of the integrins have been associated with cancer, which prompted the development of PET radioligands, e.g., the labeled version of the RGD motif binding to α_v_β_3_ [51]. Some integrins have also been shown to be involved in inflammation and fibrosis, for example, α_v_β_6_. Both α_v_β_3_ [52,53] and α_v_β_6_ [54,55] have thus received interest as biomarkers for fibrogenesis. However, apart from fibroblasts, both α_v_β_3_ and α_v_β_6_ are expressed on other cell types in fibrotic lesions, such as macrophages [56] and activated endothelial [57] and epithelial cells [55]. Neither α_v_β_3_ nor α_v_β_6_ may thus be a pure fibroblast marker in the context of imaging.

α_v_β_3_-targeted PET probes, originally developed for cancer detection, have been repurposed and evaluated in preclinical models of fibrosis in liver [32,33] and lung [34]. These promising preclinical results indicate that α_v_β_3_ PET imaging could be used to localize fibrotic lesions with ongoing activity. Importantly, α_v_β_3_-targeting PET tracers are already widely available for clinical use, and studies in human cohorts with fibrotic disease could thus be performed with existing technology. However, it is still unclear which cell types are the primary target for α_v_β_3_ imaging in fibrotic lesions, potentially making interpretation of the signal challenging.

Several α_v_β_6_-targeted PET tracers have similarly been developed for detection of cancers. Similar as for α_v_β_3_ above, some of these have been evaluated also in fibrotic disease, mainly lung. Preclinical data on α_v_β_6_-based PET tracers in animal models of lung fibrosis are quite lacking in the literature, with only a few reports available [35]. Instead, there are several published clinical studies on α_v_β_6_ PET in lung fibrosis. Two different radiolabeled peptides, A20FMDV2 [36] and R01-MG-F2 [37,38], both radiolabeled with fluorine-18 (t_1/2_ = 109.7 min; β^+^-emission of 97%), have been evaluated in patients with idiopathic pulmonary fibrosis (IPF), in comparison with healthy cohorts. Both tracers demonstrated increased binding in the lung of patients with IPF, consistent with α_v_β_6_ immunohistochemistry. Interestingly, [^18^F]FB-A20FMDV2 was also used to estimate the target engagement of the α_v_β_6_ inhibitor GSK3008348 in the lung of patients with IPF, demonstrating around 20% drug occupancy at α_v_β_6_ after inhalation [39].

#### 2.1.2. Platelet-Derived Growth Factor Receptor Beta (PDGFRβ)

PDGFRβ is a classical pericyte marker and, thus, involved in many crucial processes during development and adulthood in both health and disease. Pericytes or their counterpart stellate cells are important contributors of collagen-producing myofibroblasts in many tissues and have thus been suggested as suitable markers for fibrogenic processes. Activated hepatic stellate cells (HSCs) are the main culprit in fibrotic collagen deposition in the liver [8]. PDGFRβ strongly upregulated on activated HSCs [58,59,60] is involved in the regulation of fibrogenesis [61], and its expression levels correlate with the fibrosis degree in liver fibrotic disease [60]. Importantly, PDGFRβ has low background expression in healthy liver and is undetectable on quiescent HSCs [58]. Similarly, PDGFRβ has been described as a marker of fibrogenic cells derived from pericytes in both lung [62,63,64] and heart fibrosis [65]. PDGFRβ has also been described as expressed on a subset of macrophages. PDGFRβ may also be expressed on activated endothelial cells in the sinusoids in, e.g., liver fibrosis.

High-affinity PDGFRβ-specific ligands suitable for PET labeling have been challenging to develop. Small-molecule ligands, e.g., repurposed tyrosine kinase inhibitors binding to the intracellular signaling C-terminal part of, e.g., the PDGF receptor family, have not been sufficiently specific for the PDGFRβ receptor subtype. PDGFRβ-specific antibodies are available but are generally too large to serve as the ideal basis for PET tracer development, given the slow biological clearance of antibodies (order of days), which in turn necessitates radiolabeling with relatively long-lived radionuclides, such as Zirconium-89 (t_1/2_ = 78 h; β^+^-emission of 23%). Antibody-based PET tracers thus carry higher radiation exposure to the scanned subject, which may limit the possibility for repeated examinations, especially in younger populations and relatively healthy cohorts. Recently, several smaller peptides with nanomolar affinity towards PDGFRβ have been discovered and are being developed for PET imaging of cancer and fibrogenic processes. Bi-cyclic peptides targeting PDGFRβ [66] have been fluorescently labeled for imaging and evaluated in preclinical models of liver [67] and kidney fibrosis [68]. A peptide with a similar binding moiety was radiolabeled with gallium-68 (t_1/2_ = 68 min; β^+^-emission of 89%) and has entered clinical studies in liver and heart fibrosis [18]. Peptides based on the Affibody molecule scaffold (58-amino-acid length in a triple-α-helix conformation) with nanomolar affinity towards PDGFRβ have been described [69]. Peptides based on this scaffold have been labeled by both fluorescence [70] and fluorine-18 [40] and successfully evaluated in preclinical liver fibrosis models for detection of fibrogenic processes and activation of HSCs. Clinical translation of a gallium-68-labeled version of the PDGFRβ-targeting Affibody molecule, named [^68^Ga]Ga-DOTA-Cys-ATH001, is ongoing.

#### 2.1.3. Fibroblast Activation Protein (FAP)

FAP is an established molecular target in nuclear medicine, with several analogues developed for PET imaging specifically in cancer in recent years. FAP is strongly and broadly expressed in tumor stroma, associated with tissue remodeling, and it potentially may correlate to tumor progression. FAP is part of the DPPIV family of peptidases. It is highly multifaceted, though, and has endopeptidase activity through a second enzymatic site. Additionally, FAP has also been shown to have function independent of its enzymatic activity, such as modulating cell proliferation, migration, and invasion of tumors [71]. Thus, FAP targeting has exhibited strong interest both from a PET diagnostic viewpoint and also as a drug target. However, its collagenase activity and association with tissue remodeling are of most interest for the topic of fibrosis detection discussed here.

FAP expression has been found on the surface of fibroblasts, e.g., in lung fibrosis, which has prompted the evaluation of available FAP-targeting radioligands also in fibrotic disease [72]. However, it is not clear if FAP expression in fibrotic lesions is indicative of progress (fibrogenesis) or resolution (fibrolysis) of disease. It was shown that the presence of FAP-expressing fibroblasts actually was protective in a mouse model of lung fibrosis [73]. FAP-/- mice exhibited worse progression of disease, indicating that FAP could be an important marker of fibrolytic fibroblasts and, thus, crucial for collagen clearance and resolution of fibrosis in this indication. This is in line with the observed collagenase activity of FAP. Regardless, detection of FAP in tissue would indicate ongoing remodeling and presumably active fibrotic disease, potentially providing a new diagnostic test in the clinic. However, understanding the exact mechanism of action is important in the context of therapy follow-up, as increased binding of FAP radioligands (based on FAP inhibitors (FAPIs)) could either indicate negative (interpreted as a marker of increased fibrogenesis towards disease *progression*) or positive effect (interpreted as a marker of increased fibrolysis towards *resolution*) (Figure 2 and Figure 3A).

Gallium-68-radiolabeled FAPI analogues have recently been extensively evaluated as diagnostic imaging agents in the oncological setting, for detection of tumor stroma. FAP expression in tumor stroma is present in many cancers and has been suggested to be involved (among other things) in remodeling of the tumor environment. As FAP is a collagenase, its enzymatic activity is crucial in collagen degradation, which may assist in tumor growth. However, FAP has many other suggested functions, including immunomodulation and downstream signaling, and thus, it is unclear exactly what high binding of ^68^Ga-FAPI represents. ^68^Ga-FAPI-04 has been evaluated in preclinical models of heart [41] and pulmonary fibrosis [42]. Gallium-68-radiolabeled FAPI was recently also clinically evaluated in IPF, demonstrating lesions especially in the basal parts of the lung [43]. Importantly, decreased binding was seen after intervention with nintedanib, interpreted by the authors as a decrease in fibrosis or fibrogenic activity. However, this interpretation is at odds with the previous preclinical literature indicating the presence of FAP as a protective mechanism in lung fibrosis, presumably through its fibrolytic activity. Furthermore, it is unclear how much of the lung binding seen in this study was specific in nature, as non-specific accumulation of PET tracers is routinely found in the basal regions of the lung simply through gravitational effects [74]. These effects are even more pronounced in individuals with high BMI and increase with the duration of lying in the prone position. It is not clear if these factors were controlled in this study. Gallium-68-labeled FAPI analogues have also been evaluated clinically in other indications, such as intestinal [44], heart [45], and renal [46] diseases. Some of the aforementioned studies are small or case reports and thus inconclusive at the moment. However, given the widespread and growing availability of FAPI PET radioligands, it is expected that larger clinical imaging trials in fibrotic diseases are upcoming.

### 2.2. Summary: Imaging of Fibrogenesis and Fibrolysis

The development and evaluation of PET tracers for detection of remodeling and collagen deposition and clearance in fibrotic lesions is an active field with rapid progress. The published literature above is recent, and while the current volume of literature is limited on this topic, it is expected that many reports will be forthcoming in the coming years. So far, no direct comparison between any of the described tracers has been performed, but theoretically, their binding should reflect active remodeling, such as fibrogenesis or fibrolysis, and not established collagen depositions.

Several clinical imaging trials using, e.g., PDGFRβ, FAP, and integrins in cohorts with fibrotic diseases are available in clinical trial databases. It is, therefore, expected that the value of PET tracers targeting fibrotic lesions with active remodeling will be better understood with regards to their mode of action in the near future.

## 3. Imaging of the Fibrotic Scar

Techniques for non-invasive detection of the fibrotic scar (e.g., the pathological ECM deposition) in tissue have been well researched for decades, and several methods are even implemented in routine clinical assessments. In the context of liver fibrosis, US and MRI can be used to measure tissue stiffness using transient elastography (TE) and magnetic resonance elastography (MRE), respectively [75]. Tissue stiffness is further associated with fibrosis degree, especially in high-grade liver fibrosis. The drawback of TE is that it is operator dependent. Furthermore, both TE and MRE lack specificity and sensitivity at low fibrosis degrees. CT is routinely used to detect lung lesions and can be used to measure density changes that are the result of local fibrosis deposition [76]. Potentially, nuclear medicine-based imaging of fibrosis may provide increased sensitivity and quantification, despite the burden associated with radiation dose.

### Targets and Radioligands to Detect the Fibrotic Scar

Collagen, fibronectin, and elastin ECM proteins can serve as biomarkers that can be targeted in molecular imaging of fibrosis, providing direct identification of the fibrotic tissue. It might present an advantage over other biomarkers, such as integrins, metalloproteinases, angiotensin-converting enzyme, and blood coagulation factors, since it may allow quantification of the extent of the fibrosis, reflecting the status of fibrotic tissue development. Thus, as a key constituent of fibrotic tissues, it may provide the most accurate and direct assessment of the status of fibrosis. The accurate quantification is crucial for the assessment of the progression and response to treatment. In this context, PET provides an advantage over SPECT due to the possibility of quantification of the accumulated radioactivity concentration as a measure of fibrotic tissue. Moreover, PET is a more sensitive (100–1000-fold) technique with higher resolution and a shorter acquisition time. Given the amount of literature on the topic of collagen imaging and the maturity of this field, we will go into deeper detail on the structure and mechanism of these radioligands.

Development of an imaging agent with a specific binding affinity to the target is of paramount interest. It is important to assure low non-specific binding to normal tissue, fast blood clearance, and washout from healthy organs in order to provide sufficient image contrast and thus unambiguous detection of target tissue. The corresponding characteristics can be modulated by the size, lipophilicity, and charge of the tracer molecule.

Collagelin, an 18-amino-acid residue cyclic peptide (c[CPGRVMHGLHLGDDEGPC]) with micromolar affinity to type-I and type-III collagen, was identified from the bacterial peptide library by Muzard et al. [15]. It presumably binds to the triple-helix structure of collagen and, thus, has the potential of highly specific imaging. It was successfully labeled with ^99m^Tc for SPECT and demonstrated in vivo binding to collagen in a lung fibrosis mouse model. Three more collagelin [7,15,16,17] analogues comprising different derivatives of 1,4,7-triazacyclononane-N,N′,N″-triacetic acid (NOTA) and labeled with ^68^Ga were developed. The common feature of the analogues is PEG_2_ linker that was introduced to conjugate peptide moiety to the chelator one and to distance the chelator moiety from the binding site, as well as to modulate the pharmacokinetics of the tracers. The analogues differed in the structure of the chelator moiety, resulting in a difference of the total charge of the Ga-complex moiety that led to the two–threefold lower uptake of the positively charged analogue in liver, spleen, and somewhat in kidney in healthy rodents. Despite low affinity in the micromolar range (K_d_ of 2.3 ± 0.8 μM and 2.1 ± 0.9 μM, respectively, for [^68^Ga]Ga-NO2A-Col and [^68^Ga]Ga-NODAGA-Col), the analogues might be useful imaging agents due to the high micromolar concentration of collagen I during active fibrosis providing sufficiently high binding potential and sensitivity of the imaging. The drawback of these analogues comprising methionine amino acid residue [16,17] was their inclination for oxidative radiolysis. The substitution of methionine with its stable isoelectric analogue, norleucine, improved the stability of the agent, while maintaining favorable organ distribution in healthy rodents and binding in fibrotic liver frozen sections [7].

A series of analogues based on a short cyclic peptide sequence (16 AA) with affinity to collagen I and comprising various numbers (1 or 3) of the chelator moiety per peptide was developed [9,10,48]. Five analogues (CBP1, CBP3, CBP5, CBP6, and CBP7) were labeled with ^64^Cu and demonstrated a collagen I binding affinity range of K_d_ = 1.6–14.6 µM [48]. The analogues were accumulated in the pulmonary fibrosis of a murine model in vivo. CBP7 was found superior in terms of specific high uptake in the target tissue, low retention in off-target organs, and high metabolic stability. The accumulation of CBP7 correlated with the stage of pulmonary fibrosis. CBP7 was then labeled with ^68^Ga to result in two more analogues, CBP8 (with L-Cys at position 4) and CBP12 (with D-Cys at position 4), that demonstrated specific and quantifiable uptake in two mouse models of pulmonary fibrosis [9]. CBP8 had stronger affinity (K_d_ = 2.1 ± 0.1 µM) with considerably more intense binding to newly formed collagen in active disease as compared to the binding to mature collagen found in skin and bone. The observation was attributed to the interaction of CBP8 with collagen monomers that are abundant in the newly forming disease but are not accessible in the manured fibrils. Thus, it is unclear if CBP8 uptake reflects binding to collagen fibrils (fibrosis) or a measure of collagen remodeling, i.e., ongoing processing and formation of collagen (fibrogenesis), or even collagen monomers exposed during fibril unwinding and degradation (fibrolysis). Regardless, tissue uptake of CBP8 is expected to relate to fibrotic processes, but improved understanding of the mode of action is required for improved interpretation of the performance of CBP8, especially in the context of therapy follow-up. The success of preclinically investigated CBP8 led to the clinical trial (NCT03535545) in patients with idiopathic pulmonary fibrosis (IPF) using ^68^Ga-CBP8/PET [10]. The imaging agent was safe, well tolerated, and accumulated in the lung in concordance with anatomic distribution of IPF. It was possible to detect areas with active collagen deposition that could not be detected by CT.

A smaller nine-amino-acid residue peptide (LRELHLNNN), derived from the binding site sequence of decorin [77,78], was conjugated to either DOTA- or NOTA-based bifunctional chelators via a PEG_2_ linker, resulting in two analogues for the subsequent labeling with, respectively, ^68^Ga and ^18^F [19]. Surface Plasmon Resonance binding studies of the unconjugated and unlabeled LRELHLNNN peptide demonstrated sub-micromolar affinity (K_d_ = 170 nM) to collagen type I. This is an improvement as compared to [^68^Ga]Ga-CBP8 (2.1 µM) [9] [10,48,49] and collagelin (1.5–2 µM) [15,16,17,47,79], but direct comparison should be taken with caution, as different assays were used for affinity assessment for the different tracers. The binding of radiolabeled peptide correlated with the fibrosis grade in liver biopsies, as assessed by Sirius Red staining of collagen. The ex vivo organ distribution studied in mice demonstrated low background uptake in the organs of interest, such as heart, liver, and lung for both analogues. However, somewhat higher uptake and longer retention in the kidney and small intestine was observed for the ^18^F-labeled counterpart. The faster clearance of the ^68^Ga-labeled counterpart from the kidney presents a considerable advantage in terms of the possibility to image collagen type I in kidney, which might not be possible with the 18F-labeled counterpart [19] or CBP8 [10,48,49] and collagelin analogues [15,16,17,47,79]. However, the agent showed low in vivo stability in rodents, with only 10–15% of the intact fraction after 60 min.

Thirteen-amino-acid residue peptide (CBP1495) functionalized with Gly-(D)Ala-Gly-Gly sequence for chelating of ^99m^Tc was reported to quantify imaging of collagen I [50]. It was hypothesized that it bound to the repeating GPO triplets in collagen since CBP1495 displayed high affinity to a mimic peptide (GPO)_9_. Moreover, no binding of the scrambled analogue (RP1495) of the same constitution, but different amino acid residue order, could be observed. The in vivo organ distribution in rat models of pulmonary and hepatic fibrosis showed accumulation in the affected areas with high image contrast.

## 4. Summarizing Remarks

Improved markers of fibrosis degree would be a welcome addition to the clinician’s arsenal of diagnostic tests during fibrosis staging. For example, nuclear imaging scanning could conceivably be used as a second-line imaging technique when readouts of the fibrosis grade from, e.g., TE in combination with peripheral biomarkers is unclear or difficult to interpret. This is similar to the use of, e.g., PET in oncology, where this technique, although expensive and conferring a radiation dose, may be cost effective in some patient groups. The assessment of the PET marker value for the fibrogenesis, fibrolysis, and fibrotic scar in the clinical diagnostic setting may take many years and rigorous clinical studies. In the shorter timeframe, these techniques have the potential to become rapidly incorporated in experimental interventional studies, serving as biomarkers to assess drug effects. Imaging technologies for reliable detection and quantification of fibrogenesis and fibrolysis are expected to have a direct impact on anti-fibrotic drug development, by providing novel assessments on molecular changes preceding changes in fibrosis degree. Such technologies will hopefully provide biomarker readouts in much shorter timeframes (weeks/months) compared to months/years.

Furthermore, imaging markers of fibrogenesis may provide clinicians with complementary information to the currently available TE and MRE assessments of fibrosis degree. Scanning for fibrogenesis markers may identify those patients who are expected to progress to higher fibrosis stages, and those who are stable. This can, thus, govern the selection of suitable novel therapies, as such become available in the clinic.

## Figures and Tables

**Table 1:** Imaging agents discussed in Section 2 and Section 3.

Imaging Agent Imaging Technique	Target, Organ, and Study Type	Refs.
**Fibrogenesis and Fibrolysis**		
[^99m^Tc]-cRGD/SPECT	α_v_β_3;_ liver; preclinical models of fibrosis	[32]
[^18^F]-alfatid/PET	α_v_β_3;_ liver; preclinical models of fibrosis	[33]
[^18^F]-FPP-RGD_2_/PET	α_v_β_3;_ lung; preclinical models of fibrosis	[34]
[^18^F]FB-A20FMDV2/PET	α_v_β_6_; lung; preclinical models of fibrosis	[35]
[^18^F]FB-A20FMDV2/PET	α_v_β_6_; IPF; clinical study	[36]
[^18^F]FP-R01-MG-F2/PET [^68^Ga]-NODAGA-R01-MG/PET [^64^Cu]-DOTA-R01-MG/PET	α_v_β_6_; IPF; clinical study	[37,38,39]
[^68^Ga]Ga-BOT5035/PET	PDGFRβ; liver; preclinical study	[18]
[^18^F]-TZ-Z0959/PET	PDGFRβ; liver; preclinical study	[40]
[^68^Ga]-FAPI-04/PET	FAP; heart, lung; preclinical study	[41,42]
[^68^Ga]-FAPI-04/PET	FAP; lung; clinical study	[43]
[^68^Ga]-FAPI/PET	FAP; intestines; clinical study	[44]
[^68^Ga]-FAPI-04/PET	FAP; heart; clinical study	[45]
[^68^Ga]-FAPI-46/PET	FAP; kidney; clinical study	[46]
**Fibrotic scar**		
[^99m^Tc]-collagelin/SPECT	Collagen I/III; preclinical study	[15]
[^68^Ga]Ga-NO2A-Col/PET [^68^Ga]Ga-NODAGA-Col/PET [^68^Ga]Ga-NO2A-[NLe^13^]-Col	Collagen I/III; preclinical study	[7,15,16,17]
[^64^Cu]-NOTA-Collagelin/PET	Collagen; preclinical study	[47]
[^64^Cu]-CBP1/PET; [^64^Cu]-CBP3/PET; [^64^Cu]-CBP5/PET; [^64^Cu]-CBP6/PET; [^64^Cu]-CBP7; [^68^Ga]-CBP8/PET	Collagen I; preclinical study	[9,48]
[^64^Cu]-CBP8/PET	Collagen I; IPF; clinical study	[10,49]
[^18^F]AlF- LRELHLNNN/PET [^68^Ga]Ga-LRELHLNNN/PET	Collagen I; preclinical study	[19]
[^99m^Tc]-CBP1495/SPECT	Collagen I; preclinical study	[50]

## Data Availability

Data sharing is not applicable.
